# The Oral–Gastric Microbial Axis in Gastric Cancer: Mechanisms Underlying Development and Progression

**DOI:** 10.3390/cancers18060977

**Published:** 2026-03-18

**Authors:** Lin He, Xiao Yu, Ding-Hong Xiao, Hai-Yan Zhang, Lian-Jun Xing, Zhi-Dong Liu

**Affiliations:** Department of 2nd Gastroenterology, Longhua Hospital Shanghai University of Traditional Chinese Medicine, No. 725, Wanping South Road, Xuhui District, Shanghai 200032, China; 72023020@shutcm.edu.cn (L.H.); richie_xiao@163.com (X.Y.); fxl_625@163.com (D.-H.X.); jacy402@126.com (H.-Y.Z.)

**Keywords:** gastric cancer, oral–gastric microbial axis, microbiota dysbiosis, microbial metabolites, tumor microenvironment

## Abstract

The etiology of gastric cancer (GC) is increasingly defined by the complex interplay within the oral-gastric microbial axis. This conceptual shift extends beyond the classical *Helicobacter pylori* (*H. pylori*) model. Instead, it encompasses a broader polymicrobial network. Mechanisms underlying ectopic colonization of oral pathobionts are examined alongside their synergistic contributions to mucosal dysbiosis. Remodeling of the tumor microenvironment is discussed through the analysis of critical functional modules, including biofilm formation, metabolic reprogramming, and immune dysregulation. Carcinogenesis is reportedly promoted by specific genotoxic metabolites and perpetuation of chronic inflammation. Diagnostic capabilities are evaluated with a focus on noninvasive biomarkers, where integration of artificial intelligence for risk stratification is identified as a transformative tool for early detection. Furthermore, therapeutic perspectives are expanded by evidence linking microbial composition to the efficacy of immune checkpoint inhibitors and chemotherapy. Strategies for prevention and treatment are proposed based on restoration of microbial homeostasis. Collectively, a roadmap for translating microbiome research into personalized clinical practice for gastrointestinal malignancies is provided by this review.

## Simple Summary

Gastric cancer remains a leading cause of cancer-related death worldwide. While *Helicobacter pylori* is a well-known risk factor, emerging evidence suggests that broader imbalances in the microbial communities of the mouth, stomach, and gut are significantly associated with disease progression. This article explores the ‘oral-gastric-gut axis’, explaining how specific bacteria and the substances they produce are linked to the disruption of the body’s immune system and metabolism, potentially facilitating tumor growth. We aim to clarify how declining bacterial diversity and expansion of harmful microbes contribute to inflammation and cancer development. These findings are significant, as they identify consistent microbial patterns that could serve as new, noninvasive tools for early diagnosis. Ultimately, understanding these complex interactions provides a foundation for developing novel therapies that target the microbiome to prevent or treat gastric cancer more effectively.

## 1. Introduction

Gastric cancer (GC) is ranked as the fifth most common malignancy worldwide and the fourth leading cause of cancer-related mortality. Approximately one million new cases and over 780,000 deaths are reported annually [1]. High incidence rates are observed primarily in East Asia, specifically within China, Japan, and South Korea. It is estimated that China alone accounts for approximately 44% of incident cases and 49% of deaths worldwide [2]. Although a gradual decline in global incidence has been noted, GC continues to represent a major public health burden in the Asia–Pacific region due to population aging and persistent environmental exposures [3,4]. A multifactorial etiology has been recognized, in which genetic susceptibility, environmental exposures, dietary patterns, and *Helicobacter pylori* (*H. pylori*) infection are implicated. Among these factors, modifiable risks such as high salt intake, smoking, excessive alcohol consumption, and obesity have been associated with a substantially increased risk of disease. Furthermore, non-modifiable determinants, including family history, age, and sex, have been shown to contribute to GC susceptibility [5]. Collectively, these influences contribute to a heterogeneous landscape of GC pathogenesis. Consequently, the need for targeted investigation of additional oncogenic mechanisms is underscored.

The microbiota is increasingly recognized as a key modulator of human health, and evidence linking microbial communities to carcinogenesis across multiple cancer types has continued to accumulate. Although *H. pylori* is established as the primary carcinogenic bacterium in GC, it is suggested by emerging data that other gastric microbial taxa contribute to tumor initiation and progression. These contributions are mediated through immune dysregulation, metabolic reprogramming, and chronic inflammation [6]. Significantly, oral and intestinal microbial communities have been implicated in the risk of GC. Oral bacteria translocated to the stomach via swallowing or reflux may interact with resident gastric microbiota. This interaction establishes an oral–gastric microbial axis, which influences gastric microbial stability and potentially drives carcinogenesis [7]. Unlike the broader oral-gut network, where the stomach functions primarily as a transit corridor, this specific axis identifies the gastric niche as a definitive site for active microbial colonization. Consequently, this concept updates the traditional *H. pylori* model by emphasizing a synergistic polymicrobial etiology rather than a solitary infection.

**Figure 1:**
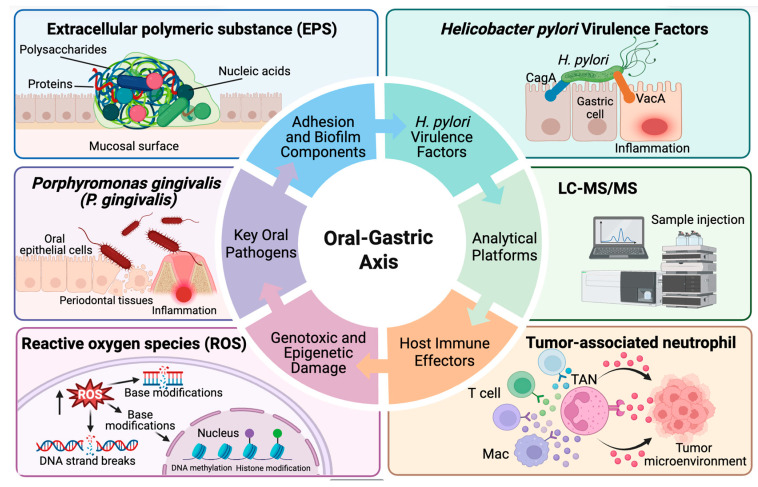
Schematic representation of the oral–gastric microbial axis in gastric cancer (GC).

In this review, the association of microbiota with GC development is examined, with particular emphasis placed on the emerging concept of the oral–gastric microbial axis. The potential roles of this axis in GC pathogenesis are summarized, and implications for prevention and therapeutic strategies are discussed (Figure 1).

Six core modules are illustrated in the inner ring: adhesion and biofilm components, *H. pylori* virulence factors, analytical platforms, host immune effectors, genotoxic and epigenetic damage, and key oral pathogens. Representative examples for each module are shown in the outer panels, including extracellular polymeric substances (EPS), *H. pylori*, LC–MS/MS, tumor-associated neutrophils (TANs), reactive oxygen species (ROS), and *Porphyromonas gingivalis* (*P. gingivalis*).

## 2. The Oral–Gastric Axis Concept and Gastric Specificity

### 2.1. Anatomical and Physiological Basis of Oral-to-Gastric Transmission

Although the oral cavity and gastrointestinal tract are anatomically distinct, functional continuity is maintained through habitual swallowing of saliva. By this mechanism, oral microorganisms are delivered to the esophagus and stomach. While the highly acidic gastric milieu is generally considered an impediment to microbial survival and sustained colonization under physiological conditions, viable microbial translocation is increasingly supported by evidence when this barrier is compromised [8]. Hypochlorhydria, particularly the condition induced by chronic proton pump inhibitor (PPI) therapy, is recognized as a major driver of this disruption. In a prospective interventional study, an increased proportion of gastric sequencing reads was attributed to oral sources following 8 weeks of PPI administration. Furthermore, the number of identical oral–gastric strain pairs was approximately doubled. These data collectively indicate that large-scale microbial translocation is facilitated by an elevated intragastric pH [9,10].

Complementing these host-driven alterations, intrinsic capacities that permit survival under gastric stress appear to be possessed by several oral pathogens. It is suggested by experimental findings that amino acids are metabolized by *Fusobacterium nucleatum* (*F. nucleatum*) and *P. gingivalis* to generate ammonia and butyrate. Consequently, local pH is increased, and bacterial persistence is enhanced. Furthermore, multispecies biofilms are formed by numerous oral taxa, and collective resistance to acid stress and host immune clearance has been reported within these structures [11].

At the molecular level, specific adaptations that facilitate mucosal seeding have been identified. For instance, the synthesis of membranes enriched with erucic acid by *F. nucleatum* via the enoyl-CoA hydratase-related protein FnFabM has been reported; through this mechanism, survival is observed even at pH 1.5 [12]. Once the acidic barrier is bypassed, stable attachment may be enabled through ligand-directed adhesion. Expression of TMPC surface protein, which binds Annexin A2 on gastric epithelial cells, has been demonstrated in *Streptococcus anginosus* (*S. anginosus*). Through this interaction, epithelial adherence is enhanced, mitogen-activated protein kinase (MAPK) signaling is activated, and barrier integrity is compromised, thereby establishing an inflammatory niche [13]. Collectively, pharmacologically induced hypochlorhydria, lipid-mediated acid resistance, and receptor-specific adhesion are inferred to constitute a coordinated pathway by which oral microbiota migrate to the stomach, persist, and remodel the gastric ecosystem.

### 2.2. Enrichment of Specific Oral Pathogens in GC Tissues

Methodological advances in metagenomics have indicated that the gastric microbiome of patients with GC increasingly resembles the oral microbiome. This phenomenon is often termed oral–gastric convergence. In a 2024 study spanning multiple stages of GC, an overall reduction in microbial α-diversity was reported alongside the selective enrichment of oral-associated taxa. Specifically, *Schaalia odontolytica* and *Streptococcus cristatus* were detected at higher levels in gastric fluid from patients with low-grade dysplasia and GC. These findings suggest the potential utility of these taxa as early indicators of oral-to-gastric colonization [14]. Consistent patterns were observed in a longitudinal cohort from Korea, where progression from atrophic gastritis to cancer was accompanied by increasing overlap between salivary and gastric communities. Notably, more than 60% of dominant genera were reportedly shared at later stages [15]. Within these altered communities, elevated abundance of *Aggregatibacter* was identified and independently validated as a marker of poor prognosis [14].

Among translocated taxa, *F. nucleatum* and *P. gingivalis* are most consistently implicated. In parallel clinical surveys, *F. nucleatum* was reported in 70–90% of *H. pylori*–negative GC biopsies and is proposed as an independent microbial risk factor [16]. Mechanistically, Wnt/β-catenin and MAPK signaling are activated by intratumoral *F. nucleatum*, thereby potentially facilitating tumor proliferation and invasiveness. Concurrently, protumorigenic neutrophil recruitment is linked to NF-κB activation [17,18]. The tumor microenvironment is likewise potentially influenced by *P. gingivalis* through immunosuppression and pathway modulation. PD-L1 expression in gastric epithelial cells is upregulated by virulence determinants such as gingipains and lipopolysaccharide (LPS), with consequent inhibition of T cell–mediated antitumor immunity [19]. Metastatic potential is further facilitated by outer membrane vesicles released by *P. gingivalis*. These vesicles are described as carrying small RNAs, specifically sRNA23392, which target desmocollin-2 (*DSC2*) [20].

Documentation of oral-associated pathobionts in GC tissues extends to organisms such as *Prevotella intermedia*. Cellular proliferation and epithelial–mesenchymal transition have been shown to be accelerated in vitro by isolates of this bacterium from tumors through the upregulation of perilipin 3 (PLIN3). Furthermore, higher intratumoral abundance is associated with advanced stage and poorer survival [21]. Distinct colonization patterns are observed in proximal (upper-third) gastric tumors, where an oral-associated module dominated by *Veillonella parvula* and *Streptococcus oralis* was identified. The presence of these taxa in *H. pylori*–negative cancers is associated with reduced overall survival, a finding that suggests a more aggressive molecular phenotype [22]. Based on these cumulative data, it was proposed in a 2025 expert review that a reproducible microbial signature linked to elevated GC risk is constituted by declining community richness combined with the selective expansion of proinflammatory taxa, such as *S. anginosus* and *F. nucleatum* [23].

### 2.3. Connection to the General Oral-Gut Microbiota Axis

Although the oral–gastric link is proposed as a distinct carcinogenic pathway, it is likely embedded within the broader framework of the oral–gut microbiota axis. This axis is described as a bidirectional regulatory system in which communication and translocation occur between oral and gut microbiomes via microbial dissemination and signaling. Viable microbial translocation and functional interactions between these communities are increasingly supported by evidence, despite the anatomical separation of the oral cavity and the gastrointestinal tract [8].

The relationship between oral and gut environments is characterized by dynamic interchange. Colonization of the gut by oral microbes swallowed with saliva is observed, particularly when epithelial barriers are compromised, resulting in disrupted microbial stability [24]. In the reverse direction, the composition and resilience of oral communities may be perturbed by the reflux-associated transfer of intestinal microbes to the oral cavity, specifically during gastroesophageal reflux. Furthermore, translocation is reported in association with dietary exposures, systemic diseases including diabetes, and antibiotic therapy [25]. Evidence of shared mechanisms shaping the tumor microenvironment along the digestive tract is provided by the observation that oral pathogens enriched in GC, including *F. nucleatum*, are frequently enriched in the gut microbiota of patients with colorectal cancer [26].

These connections are elucidated largely through methodological advances. Metagenomics and metabolomics are relied upon for most investigations of the oral–gut microbiota axis. Specifically, 16S rRNA gene sequencing provides a cost-effective taxonomic overview, whereas shotgun metagenomics yields superior resolution for species-level identification and functional gene profiling. Despite these analytical capabilities, microbiome profiling in gastric research is frequently limited by inherent methodological challenges, particularly significant host DNA interference and low microbial biomass. Concurrently, microbial metabolites with potential effects on host physiology are identified via metabolomic profiling, a process often performed using liquid chromatography–mass spectrometry (LC–MS) [27]. For instance, alteration of short-chain fatty acid (SCFA) production and bile acid metabolism by oral microbes following gut colonization is indicated by integrated metagenomic and metabolomic analyses. Through these metabolic shifts, intestinal inflammatory responses are potentially modulated [28]. Further support for the oral–gut microbiota axis as a relevant conceptual framework for GC research is provided by metagenomic tracking, which suggests that oral bacteria detected in GC tissues overlap with taxa commonly observed in the gut microbiome [29].

## 3. Gastric Dysbiosis and Microbial Interactions

### 3.1. Progressive Alterations of Gastric Microbiota During Carcinogenesis

Marked ecological shifts within the gastric microbial community during gastric carcinogenesis are indicated by growing evidence. Relative to healthy individuals, reduced microbial diversity is repeatedly observed in GC alongside the expansion of taxa associated with carcinogenic processes. Specifically, significant enrichment of oral-associated genera, including *Fusobacterium*, *Peptostreptococcus*, and *Streptococcus*, is documented in GC tissues by 16S rRNA gene sequencing studies. In contrast, healthy tissues are reported to be predominantly colonized by *Lactobacillus* and *Lactococcus* [30]. These tissue-level observations are further supported by large-scale clinical datasets. In the DELIVER clinical trial conducted in Japan, fecal profiles from 476 patients with advanced GC and 106 healthy controls were analyzed. The patient cohort was characterized by increased relative abundance of *Streptococcus*, *Lactobacillus*, and *Odoribacter*, whereas controls were enriched for *Bifidobacterium* and *Anaerostipes* [31]. Collectively, these patterns suggest that key metabolic and immune pathways are closely associated with the shifts in tumor-associated microbial communities. Consistent with this premise, enrichment of bacterial genes involved in amino acid metabolism and nucleotide transport is indicated by functional predictions derived from metagenomic analyses, a finding that suggests metabolic adaptation within the tumor microenvironment [32].

The features of gastric dysbiosis at both taxonomic and functional levels have been further clarified by recent integrative studies. A consistent bloom of *Lactobacillus* and *Streptococcus*, accompanied by depletion of *Rothia* and *Porphyromonas*, was confirmed by a 2024 meta-analysis of 33 case–control cohorts (*n* = 4829) [33]. The magnitude of these effects was reported to vary by geography. For instance, *Helicobacter* and *Streptococcus* were particularly elevated in Korean cohorts. Variation was also noted by specimen type, as more pronounced shifts were exhibited by mucosal samples compared to fecal samples [33].

Enrichment of tumor-stage gastric tissues for oral-associated genera, including *Veillonella* and *Peptostreptococcus*, alongside proinflammatory *Erysipelotrichaceae*, was reported in an updated synthesis published in Gut. Conversely, health-associated *Lactobacillales* are diminished [34]. Complementing these taxonomic changes, upregulation of microbial functional pathways is revealed by metagenomic reconstruction. These pathways include urease activity, LPS biosynthesis, and nitrate reduction, all of which may aggravate mucosal inflammation and facilitate DNA damage [34]. Mechanistic insights are further deepened by the application of advanced computational approaches to gastrectomy-derived shotgun metagenomes, such as the PredCMB gene–metabolite pipeline. Through these methods, selective enrichment of metabolites linked to polyamine biosynthesis and sulfur metabolism is predicted. Mechanistic clues are provided by these findings regarding how dysbiosis may contribute to accelerated tumor growth and enhance redox-stress tolerance within the gastric microenvironment [35].

### 3.2. Synergistic Interactions Between H. pylori and Oral Bacteria

Although complex community-level interactions are involved in shaping the gastric microbiome, *H. pylori* is regarded as a primary ecological driver that establishes molecular and immunological conditions conducive to carcinogenic progression and potential microbial synergy. A central determinant of pathogenicity is the virulence factor CagA, which is translocated into gastric epithelial cells, where a pro-tumorigenic program is orchestrated. Through aberrant activation of multiple downstream pathways, including MET, PI3K/AKT, NF-κB, and Wnt/β-catenin, proliferation and migration are promoted by CagA signaling while resistance to apoptosis is increased [36]. Crucially, these specific oncogenic effects are fundamentally restricted to CagA-positive strains. Because CagA-negative variants naturally lack this potent virulence factor, they do not possess the same capacity to hijack host signaling networks or drive pronounced malignant transformation. This fundamental distinction underscores the importance of evaluating strain-specific pathogenic potential in clinical contexts. Concurrently, a pro-carcinogenic inflammatory milieu is induced by *H. pylori*–mediated immune dysregulation, a state characterized by enhanced Th17 activity and elevated secretion of IL-6 and IL-10. In animal and zebrafish models, a reduction in central memory regulatory T cells (Tregs) is observed during disease progression, whereas early-stage responses are marked by heightened Th17 activity [37]. This dysregulation is further amplified by epigenetic remodeling. Specifically, DNA methylation–mediated silencing of tumor-suppressive microRNAs, such as miR-210, is induced by *H. pylori*. Consequently, oncogenic targets such as *STMN1* and *DIMT1* are upregulated, and aberrant proliferation of gastric mucosal cells is driven [38]. Furthermore, the TLR/MyD88 signaling pathway is identified as a critical mediator of *H. pylori*–induced immune activation and tumorigenesis through the modulation of tumor-associated immune-cell phenotypes and cytokine profiles [39].

Carcinogenesis is also promoted by *H. pylori* through mechanisms extending to non-canonical pathways, as indicated by recent structural and functional analyses. A pivotal regulatory element is identified in the phase-variable outer membrane protein OipA. The efficiency of the type IV secretion system (T4SS) is reported to increase when OipA is in the “on” state. Conversely, reduced CagA phosphorylation and altered bacterial morphology are observed when OipA is “off”. These findings position OipA as a key checkpoint that controls effector translocation and epithelial responses [40]. Structural insights are further provided by high-resolution cryo-electron microscopy (cryo-EM), which has resolved key architectural features of the secretion apparatus. These features include CagX-enriched subdomains within the outer membrane core complex. The structural flexibility of these domains is suggested to influence the efficiency of CagA delivery, thereby contributing to the strain-specific oncogenic potential observed clinically [41].

Mitochondrial injury is independently induced by the vacuolating cytotoxin VacA, and a mitophagy response is consequently triggered. Although initially protective, chronic activation of this pathway is linked to deleterious outcomes that culminate in autophagic cell death and genomic instability [42]. Exploitation of this pathway by CagA is also observed. Specifically, mitophagy is promoted to suppress NLRP3 inflammasome activation and apoptosis, an effect that sustains a persistent inflammatory niche favoring tumor survival [43]. Prolonged oncogenic signaling is further ensured by the upregulation of the RNA-binding protein AUF1, which is reported to prevent lysosomal degradation of intracellular CagA [44]. These findings collectively support a coordinated pathogenic strategy in which virulence gene regulation, secretion-system plasticity, and manipulation of host mitochondrial quality control converge to sustain chronic inflammation. Through these processes, immune evasion is facilitated, and a permissive microenvironment for synergistic interactions with oral pathobionts is created.

### 3.3. Gastrointestinal Microecological Imbalance in GC Progression

Gastrointestinal microecological imbalance is implicated as a key factor associated with GC progression through the exacerbation of inflammatory activation, facilitation of immune evasion, and disruption of metabolic homeostasis. Enrichment of potentially proinflammatory genera, including Desulfovibrio and Escherichia, is reported in patients with GC. The association of these taxa with increased levels of proinflammatory cytokines, specifically IL-1β and IL-18, suggests that sustained inflammation represents a critical mechanism contributing to tumor initiation [45]. Impairment of intestinal mucosal barrier integrity and alteration of the Treg-to-Th17 cell balance are shown to be linked to GC-associated microbiota. Creation of an immunologically permissive microenvironment is proposed to correlate with this shift. Within this milieu, immune surveillance is attenuated, and tumor immune escape is enabled [46]. Functional gene analyses have further revealed increased abundance of genes related to hydrogen sulfide (H_2_S) production and dysregulated amino acid metabolism in the gut microbiota of patients with GC. Tumorigenesis is suggested to be facilitated by the resulting metabolites through the induction of DNA damage and activation of oncogenic signaling pathways [47].

GC-associated dysbiosis is indicated by recent multi-omics studies to extend beyond bacterial imbalance, incorporating metabolite-driven selection and shifts in viral and fungal communities. Accumulation of unconjugated secondary bile acids (SBAs), particularly deoxycholic acid (DCA), is reported in tumor mucosa. This accumulation selectively promotes the expansion of proinflammatory taxa such as *Veillonella* and *Erysipelotrichaceae*, while commensal *Rothia* is suppressed. Uptake of DCA by bacteria is demonstrated in in vitro experiments. Consequently, ribosomal transcription and amino acid metabolism are reprogrammed, a process that provides a mechanistic link between bile acid stress and nutrient pathways capable of supporting rapid tumor growth [48]. Establishment of a “bile acid amplification loop” by the expansion of the *bai* operon within GC-associated communities is suggested by a pharmacology meta-analysis. This loop sustains mucosal inflammation and promotes the progression of precancerous lesions [49]. Disruption is also reported in the fungal compartment. A consistent reduction in mycobiome α-diversity and an increased Ascomycota-to-Basidiomycota ratio in GC were described in a systematic review of gastrointestinal tumors. These data indicate that fungal antigens may further contribute to a tumor-tolerant immune milieu [50].

While bacterial and fungal alterations are central to gastric dysbiosis, oncogenic viruses act as equally important drivers of gastrointestinal malignancies. Recent evidence highlights how specific viral oncoproteins, including Hepatitis B Virus (HBV) HBx, Hepatitis C Virus (HCV) core protein, and Epstein–Barr Virus (EBV) latent membrane protein 1 (LMP1), actively hijack host signaling networks. These viral proteins fundamentally disrupt cellular homeostasis by inactivating tumor suppressors like p53 and retinoblastoma protein (Rb) and constitutively activating pro-proliferative pathways such as NF-κB and Akt, which ultimately drive chronic inflammation and metabolic dysregulation [51]. Within the specific landscape of GC, EBV infection defines a distinct molecular subtype (EBVaGC) that frequently presents as carcinoma with lymphoid stroma [52]. This unique entity exhibits a genomic profile characterized by extensive DNA hypermethylation, recurrent mutations in *PIK3CA* and *ARID1A*, and the marked overexpression of *JAK2* and PD-L1 [53]. Given its pronounced immune activation and elevated PD-L1 levels, this virus-driven subtype is increasingly recognized as a highly vulnerable target for emerging immunotherapies.

Collectively, the convergence of these alterations in bile acid metabolism and in viral and fungal populations with previously described bacterial signatures appears to generate a functionally rewired microecosystem. Within this ecosystem, genotoxic metabolites are supplied, antitumor immunity is suppressed, and the metabolic demands of proliferating GC cells are supported.

### 3.4. Multi-Omics Perspectives on the Oral-Gastric Axis

Most investigations of the oral-gastric axis currently rely on compositional sequencing methods that identify microbial dysbiosis but fail to establish mechanistic causality. Bridging the gap from correlation to causation requires the application of integrative multi-omics pipelines. Combining metagenomics with metabolomics allows researchers to map specific bacterial products, such as short-chain fatty acids and polyamines, directly to their functional roles within the gastric tumor microenvironment [27,28]. Furthermore, the incorporation of host transcriptomic and epigenetic profiling reveals exactly how translocated oral pathogens manipulate local immune responses and contribute to genomic instability [38]. The key molecular targets, signaling networks, and biological consequences elucidated by these integrated approaches are systematically summarized in Table 1.

## 4. Molecular Mechanisms Linking the Axis to Pathogenesis

### 4.1. Metabolic Reprogramming and the Role of Bile Acids

Microbial metabolites derived from oral and gut microbiota are implicated in GC progression through the modulation of host immunity, intracellular signaling, and epigenetic regulation. Among these metabolites, SCFAs and SBAs are viewed as functionally contrasting influences within the metabolic landscape. SCFAs, particularly butyrate, are generally associated with anti-inflammatory and antitumor activities. Induction of apoptosis and suppression of tumor cell proliferation are reported to be mediated by butyrate, effects achieved in part through the inhibition of histone deacetylase (HDAC) activity and consequent modulation of tumor-suppressor gene expression [54]. Furthermore, activation of SCFA-mediated antitumor signaling via G-protein-coupled receptors (GPCRs) is shown to contribute to metabolic homeostasis, a process that may confer protection against GC [55]. In contrast, protumorigenic effects are linked to SBAs such as DCA, especially when present at elevated concentrations. Attenuation of antitumor immunity by DCA through the impairment of CD8+ T cell function is suggested by available evidence. This mechanism facilitates immune evasion within the tumor microenvironment and promotes GC progression [56]. Oncogenic pathways such as NF-κB are also reported to be activated by SBAs, leading to the stimulation of proinflammatory cytokine production and increased DNA damage in host cells. These effects may collectively facilitate gastric carcinogenesis [57]. Thus, the biological impact of microbial metabolites appears to be context dependent and influenced by metabolite identity, local concentration, and host physiological state.

GC-associated metabolic reprogramming extends to polyamines, tryptophan-derived indoles, and trimethylamine N-oxide (TMAO). Upregulation of spermine oxidase (SMOX) in gastric epithelial cells is shown to be mediated by *H. pylori*. This upregulation promotes the conversion of spermine to spermidine while generating the reactive aldehyde acrolein. Accumulation of acrolein is detected in both human and murine stomachs and is linked to DNA adduct formation and accelerated malignant transformation. Reduction in tumor incidence in INS-GAS mice is notably reported following genetic or pharmacological SMOX blockade [58].

Distinct from these pro-oncogenic effects, several indole derivatives, including indole-3-lactic acid, indole-3-propionic acid, and indole-3-aldehyde, are produced by Lactobacillus species and other oral and gut commensals. Activation of aryl hydrocarbon receptor (AhR) signaling, restraint of IL-17/Th17-associated inflammation, enhancement of CD8+ T cell function, and inhibition of epithelial proliferation are reported functions of these metabolites. Together, these observations support a context-dependent tumor-suppressive role for the indole branch of tryptophan metabolism [59]. In opposition to these protective effects, the choline-derived metabolite TMAO is implicated as a protumor effector. The association of elevated gastric TMAO levels with poorer outcomes is documented, and amplification of oxidative stress and NF-κB signaling alongside compromised epithelial tight-junction integrity is suggested by mechanistic studies. This combination creates a microenvironment potentially permissive for cancer progression [60]. Collectively, these findings illustrate how diverse microbial metabolites, encompassing both oncogenic and protective species, intersect with inflammatory, genotoxic, and immune pathways to shape the trajectory of gastric carcinogenesis.

### 4.2. Modulation of the Tumor Microenvironment and Immune Responses

The tumor microenvironment is potently modulated by chronic microbial colonization, and persistent inflammation predisposing to GC can be initiated. Toll-like receptors (TLRs) are activated by virulent microbes such as *H. pylori*. Consequently, NF-κB signaling is engaged, and sustained secretion of proinflammatory cytokines, including IL-1β, TNF-α, and IL-6, is driven. Apoptosis resistance, increased cellular proliferation, and enhanced DNA damage have been linked to prolonged inflammatory exposure [61]. The immune landscape is also reshaped by chronic inflammation through alteration of the balance among Th17 cells, Tregs, and myeloid-derived suppressor cells (MDSCs). An immunosuppressive milieu is promoted by this shift; consequently, effective antitumor immunity is weakened, and immune evasion is facilitated [62].

In parallel, exploitation of specific metabolic substrates by commensals such as *Escherichia coli* is enabled by dysbiosis. Generation of genotoxic products, including reactive oxygen species (ROS) and nitrites, is subsequently observed. Cellular injury may be aggravated by these metabolites, and oncogenic mutations contributing to GC progression may be promoted [63]. Collectively, chronic inflammation and immune dysregulation are driven by microbes. Through these processes, not only is the development of precancerous gastric lesions promoted, but GC progression is also sustained via immunosuppression and metabolic disturbance.

Mechanisms extending past canonical TLR and NF-κB signaling are suggested by recent studies, in which GC-associated microbes interfere with innate immune sensing and promote the recruitment of immunosuppressive myeloid populations. The cGAS-STING and RIG-I pathways are reported to be dampened by viable *H. pylori* through the blocking of IRF3 phosphorylation. Through this mechanism, type I interferon production is reduced, and cytokine programs are biased toward a protumor Th17 profile [64]. Furthermore, remodeling of the gut microbiota by GC cells is shown to increase SCFAs turnover. This alteration is linked to the systemic expansion and gastric homing of polymorphonuclear MDSCs (PMN-MDSCs). Arginase 1 and ROS are secreted by these cells; concurrently, CD8+ T-cell cytotoxicity is suppressed, and formation of a premetastatic niche accelerating tumor growth is supported [65]. Immunosuppressive signaling may be further reinforced by microbe-associated extracellular vesicles. Delivery of PD-L1-inducing cues to macrophages and dendritic cells by exosomes from patients with GC, which are enriched in bacterial components, has been reported. As a result, antigen presentation is impaired, and checkpoint blockade efficacy is weakened [66]. Engagement of ST2 on infiltrating myeloid cells can also be affected by the dysbiosis-associated release of the epithelial alarmin IL-33. These cells are subsequently polarized toward M2-like and MDSC-like programs, an effect that enhances local immune tolerance [67]. Together, a model is supported by these mechanisms in which the oral–gut microbial axis sustains chronic inflammation while enabling multi-layered immune evasion that promotes GC progression.

**Figure 2:**
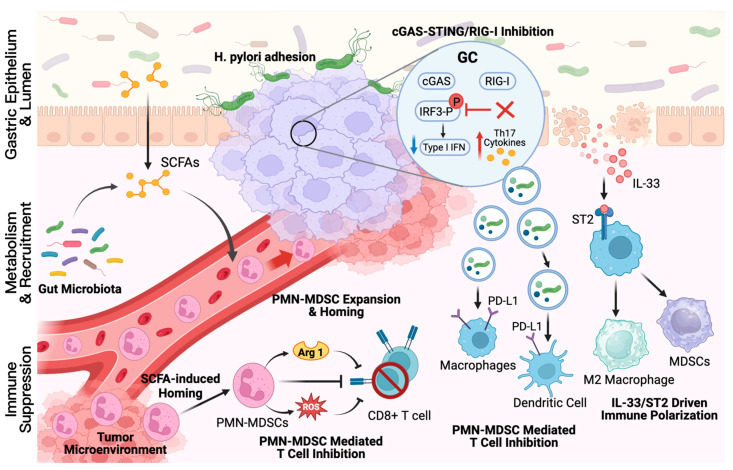
Immune microenvironment remodeling and chronic inflammation driven by the oral–gut microbial axis in GC. Red upward arrows (↑) indicate promotion, and blue downward arrows (↓) indicate inhibition.

Figure 2 outlines how the oral–gut microbial axis is associated with an immunosuppressive tumor microenvironment in GC. Top, *H. pylori* inhibits cGAS-STING and RIG-I signaling by blocking IRF3 phosphorylation, reducing type I interferon and favoring a Th17-biased cytokine profile. Bottom left, dysbiosis alters short-chain fatty acid (SCFA) metabolism and facilitates gastric homing of polymorphonuclear myeloid-derived suppressor cells (PMN MDSCs), which suppress CD8+ T cell cytotoxicity via arginase 1 (Arg1) and ROS. Bottom right, microbe-associated exosomes induce PD-L1 expression on macrophages and dendritic cells, while IL-33 engages ST2 to promote M2-like polarization and immune tolerance.

### 4.3. Microbiota-Induced Genomic Instability and Epigenetic Changes

Disruption of host genomic integrity and epigenetic regulation constitutes a second major mechanism by which carcinogenesis is influenced by the oral–gut microbiota. Genomic instability is reportedly increased by certain bacterial taxa, including *F. nucleatum* and *Prevotella*, through the induction of ROS. Consequently, DNA strand breaks and mutation accumulation are exacerbated [68]. The epigenetic machinery is also influenced by microbes, an interaction that indirectly exacerbates genomic instability. DNA methyltransferase (DNMT) activity is suggested to be modulated by specific microbes or their metabolites, including indole derivatives. Aberrant promoter methylation of tumor-related genes results from this modulation, facilitating tumorigenesis [69]. Strong correlations between microbial community shifts and dysregulated microRNA profiles in GC tissues are identified by multi-omics studies. These findings support the concept that host transcriptional programs are rewired by microbiota through noncoding RNA pathways, thereby influencing cellular transformation [70]. Microbiota-associated genotoxic stress and epigenetic remodeling thus represent important mechanisms contributing to GC initiation and progression.

Three complementary routes by which the oral–gut axis destabilizes the gastric genome and epigenome are supported by emerging evidence. Intraluminal hydrogen H_2_S levels can be increased by sulfur-reducing taxa enriched in dysbiotic stomachs, including *Desulfovibrionaceae* and *Veillonella*. H_2_S is implicated as a genotoxic stressor capable of inducing 8-oxoguanine lesions and double-strand breaks in gastric epithelial DNA. Mutational pressure is thereby imposed during malignant transformation [71]. Production of colibactin, a polyketide genotoxin, is attributed to genotoxic pks-positive *Escherichia coli* detected in upper gastrointestinal specimens. Interstrand cross-links are formed, and γH2AX foci are induced in gastric organoids by this toxin. Acceleration of dysplasia in INS-GAS mice and enhancement of chromosomal instability signatures in vivo following colibactin exposure are reported by recent work [72].

Evidence for microbiota-linked epigenetic reprogramming is likewise becoming increasingly evident. Targeted CpG methylation profiling of genes such as *TWIST1* and *DKK3* was integrated with 16S-based community indices in a 2024 case–control analysis. A marked increase in the odds of *H. pylori*–negative GC was reported in association with a high GC microbiome index combined with *TWIST1* hypermethylation. A functional association between specific taxa and DNMT-linked methylation changes is highlighted by these results [73]. Convergence of toxin-mediated DNA damage, colibactin-associated cross-linking, and microbiota-associated methylome perturbations is inferred to compromise genomic integrity and epigenetic control. Through this convergence, GC initiation and progression are accelerated.

## 5. Clinical Implications and Translational Perspectives

The identification of robust biomarkers for early detection and risk prediction is driven by the accumulating evidence linking gastrointestinal and oral microbial dysbiosis to gastric carcinogenesis. A noninvasive alternative to conventional gastroendoscopic assessment and serum tumor markers is provided by microbiome-based profiling, which offers particular value for screening and longitudinal monitoring in high-risk populations. Distinct microbial community features are exhibited in the gastric mucosa of patients with early gastric cancer (EGC) compared with individuals presenting with chronic gastritis or advanced GC. The feasibility of microbiome-enabled early detection strategies is supported by the work of Wang et al., in which a microbial classifier was developed to discriminate patients with EGC from healthy individuals and those with advanced GC [74]. Diagnostic investigations have been successfully extended beyond the gastric niche to include gut environmental signatures. Risk stratification based on gut microbiota profiles was evaluated by Zhang et al. through the construction of a random forest classifier. An area under the curve (AUC) of 0.91 was reported for differentiating GC patients from healthy controls, with *Lactobacillus* and *Megasphaera* identified as key predictive genera [32]. Quantitative frameworks for estimating lesion progression risk are further provided by algorithmic scoring systems such as the Gastric Mucosal Dysbiosis Detection Score (RGM-DT). High specificity (88.9%) in identifying high-risk patients without *H. pylori* infection was notably reported using this metric, which relies on the proportional abundance of *Bacillus* and *Veillonella* [75]. The utility of these microbial signatures is potentially enhanced by their integration with liquid biopsy markers. Salivary and serum microRNAs (miRNAs), noted for their stability, are explored alongside microbial data, with reproducible diagnostic performance indicated by systematic reviews regarding a 12-miRNA serum panel [76].

**Figure 3:**
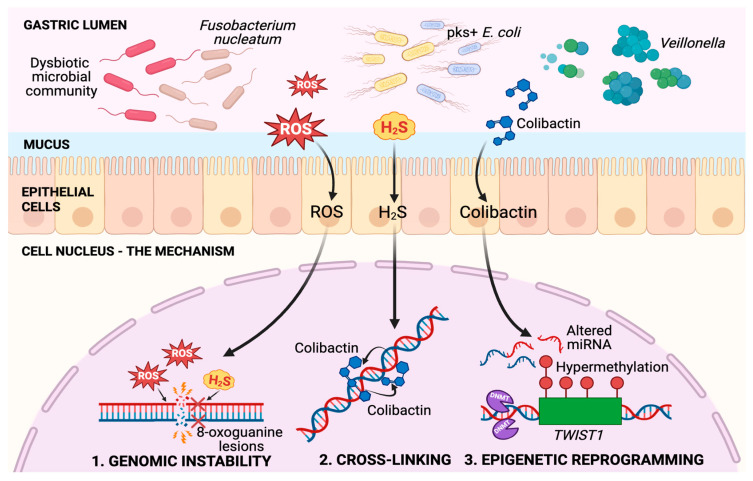
Mechanisms of microbiota-induced genomic and epigenetic instability in GC.

Figure 3 summarizes three pathways through which the microbiota contributes to genomic and epigenetic instability during gastric carcinogenesis. First, sulfur-reducing taxa and *F. nucleatum* increase H_2_S and ROS, leading to 8-oxoguanine accumulation and DNA double-strand breaks. Second, pks-positive *Escherichia coli* produce colibactin, which induces DNA interstrand cross-links and chromosomal instability. Third, microbial metabolites modulate DNA methyltransferase (DNMT) activity and noncoding RNA programs, resulting in promoter hypermethylation of tumor suppressors such as TWIST1 and aberrant microRNA profiles. Together, these alterations facilitate progression from gastric epithelium to dysplasia and carcinoma.

Parallel to these diagnostic advancements, modulation of the gastrointestinal and oral microbiome is suggested as a key therapeutic intervention to enhance the efficacy of conventional treatments. Sensitization of tumors to chemotherapy by specific probiotic strains is indicated by preclinical studies. Enhancement of capecitabine efficacy by the administration of *Lactobacillus rhamnosus* was demonstrated in a mouse xenograft model, resulting in marked tumor volume reduction and the induction of apoptosis [77]. Mechanistically, mitogenic signaling in GC cells (HGC-27) is reported to be inhibited by L. rhamnosus via the modulation of key polyamine-metabolizing enzymes [78]. Extending these preclinical observations, the clinical translation of L. rhamnosus as a prophylactic and therapeutic adjunct is gaining significant traction. By reinforcing the gastric mucosal barrier and restoring immune homeostasis, targeted probiotic administration may provide a viable strategy to impede the progression of precancerous lesions [79].

In human cohorts, improvement of gut microbial homeostasis was shown to result from combining probiotics with dietary modifications, an effect that may indirectly limit GC progression [80]. The interplay between the microbiome and immune checkpoint inhibitors (ICIs) represents a frontier in precision therapy. An unexpected association between *H. pylori* infection and increased immune-related progression-free survival was revealed by a comprehensive study involving 218 GC patients. Higher alpha-diversity and an enrichment of beneficial species, such as *Clostridium leptum* and *Ruminococcus bromii*, were exhibited by *H. pylori*-positive patients, suggesting that infection status combined with specific commensals could serve as a biomarker for immunotherapy response [81]. Potentiation of anti-PD-1 therapy efficacy by *Akkermansia muciniphila* was further demonstrated in murine models (MFC cells). This synergistic effect was characterized by inhibited tumor growth (*p* < 0.0001) and enhanced apoptosis, driven by CD8+ T-cell accumulation and a reduction in the relative abundance of potential pathogens like *Escherichia coli* [82].

Translating complex microbial features into clinically actionable tools relies heavily on artificial intelligence (AI) and machine learning. Classifiers built upon linear discriminant analysis distinguish early malignancy from healthy states with high accuracy [75]. Similarly, random forest algorithms convert the relative abundance of specific genera, such as Lactobacillus and Megasphaera, into reliable predictive scores [32]. Algorithmic systems like the RGM-DT further provide a quantitative framework to estimate progression risk based on dysbiotic signatures [76]. However, clinical implementation requires addressing challenges in reproducibility and cohort heterogeneity through rigorous validation in independent populations. This stratification facilitates personalized interventions as alternatives to broad-spectrum antibiotics. Targeted approaches using engineered bacteriophages and precision probiotics can then selectively eradicate pro-tumorigenic oral pathobionts while preserving the commensal ecosystem.

## 6. Conclusions

The conceptual framework of gastric carcinogenesis is being fundamentally expanded by the delineation of the oral-gastric microbial axis. It is increasingly recognized that GC etiology extends beyond the classical single-pathogen model driven solely by *H. pylori*. Instead, a complex polymicrobial ecosystem is implicated, wherein the ectopic colonization of oral pathobionts and their synergistic interactions with gastric residents create a pro-tumorigenic microenvironment. This review has highlighted that the disruption of the gastric mucosal barrier is not merely a consequence of infection but a cumulative result of dysbiotic networking, biofilm formation, and the continuous influx of genotoxic and inflammatory stimuli from the oral cavity.

A critical convergence of metabolic reprogramming and immune dysregulation is identified as the primary engine driving this malignant transformation. The tumor microenvironment is reshaped by specific microbial functional modules, particularly the enrichment of secondary bile acid producers, the dysregulation of polyamine metabolism, and the accumulation of pro-inflammatory metabolites. These molecular signals act in concert to sustain chronic inflammation, induce genomic instability, and facilitate immune evasion. Notably, the capacity of the microbiome to modulate responses to immune checkpoint inhibitors highlights a pivotal intersection between microbial ecology and host immunity. The finding that specific commensal signatures can potentiate the efficacy of anti-PD-1 therapies suggests that the gut microbiome acts as a distinct, modifiable organ that dictates therapeutic outcomes.

Despite these mechanistic insights, clinical translation is hindered by methodological heterogeneity and reliance on cross-sectional profiling. These observational approaches readily identify microbial associations but fail to establish causality, making it difficult to determine whether specific taxa actively drive carcinogenesis or merely colonize the altered tumor microenvironment opportunistically. Compounding these issues, gastric mucosa and fluid represent low-biomass environments highly susceptible to reagent contamination and sequencing bias. Detecting oral pathogens in the stomach also requires extreme caution due to the inevitable risk of bacterial carryover during endoscopy. To overcome these limitations, the field must transition from descriptive correlations to rigorous causal validation using germ-free models and organoid systems. Concurrently, developing noninvasive diagnostic tools relies on decoding high-dimensional microbial datasets through artificial intelligence. Machine learning algorithms effectively integrate taxonomic, functional, and host-derived markers such as microRNAs to achieve precision risk stratification. Validating these computational models across diverse geographical and ethnic cohorts remains an essential step to ensure their global applicability.

Future longitudinal studies must capture the temporal dynamics of the oral-gastric ecosystem to distinguish early microbial drivers from secondary colonizers thriving in the achlorhydric tumor microenvironment. Clarifying these shifts is essential to define the optimal window of intervention. Specifically, targeting the microbiota during the transition from chronic to atrophic gastritis, before the onset of irreversible metaplasia, could effectively halt malignant progression. To facilitate this clinical translation, standardized multi-omics pipelines are required to identify actionable therapeutic targets, including precision probiotics, dietary modulation, and fecal microbiota transplantation. Ultimately, restoring microbial homeostasis provides a compelling framework for the prevention and personalized treatment of GC.

## Figures and Tables

**Table 1:** Multi-omics mechanisms of the oral–gastric microbial axis in gastric carcinogenesis.

Omics Layer	Microbial Source & Effectors	Molecular Targets and Pathways	Biological Consequences in GC
Metabolome	*F. nucleatum* *P. gingivalis* Translocated oral taxa	Ammonia and butyrate (SCFAs) Secondary bile acids Polyamines and sulfur metabolites	↑ Local pH (supports survival) ↑ Redox-stress tolerance Alters tumor metabolic microenvironment
Host Transcriptome	*F. nucleatum* *S. anginosus* (TMPC protein) *P. intermedia*	Wnt/β-catenin and MAPK signaling PLIN3 upregulation *DSC2* suppression (via sRNA)	↑ Epithelial–mesenchymal transition (EMT) ↑ Tumor cell proliferation Associated with increased tumor invasiveness
Immune Signaling	*P. gingivalis* (Gingipains, LPS) *F. nucleatum* *H. pylori* (CagA, OipA)	PD-L1 upregulation NF-κB and TLR/MyD88 activation Cytokines: IL-6, IL-10, IL-1β	↓ T cell-mediated antitumor immunity ↑ Tumor-associated neutrophils (TANs) Facilitates Th17/Treg dysregulation
Epigenetic Modulation	*H. pylori* Associated oral pathobionts	DNA hypermethylation (miR-210) *STMN1* and *DIMT1* activation Genotoxic ROS production	Silences tumor-suppressive microRNAs Drives aberrant mucosal proliferation Linked to genomic instability

Note: ↑ indicates an increase/promotion, and ↓ indicates a decrease/inhibition.

## Data Availability

Not applicable.

## Abbreviations

The following abbreviations are used in this manuscript:

AUC	area under the curve
Arg1	arginase 1
AhR	aryl hydrocarbon receptor
cryo-EM	cryo-electron microscopy
DCA	deoxycholic acid
DSC2	desmocollin-2
DNMT	DNA methyltransferase
EGC	early gastric cancer
EPS	extracellular polymeric substances
*F. nucleatum*	*Fusobacterium nucleatum*
RGM-DT	Gastric Mucosal Dysbiosis Detection Score
GC	gastric cancer
GPCRs	G-protein-coupled receptors
*H. pylori*	*Helicobacter pylori*
HDAC	histone deacetylase
H_2_S	hydrogen sulfide
LPS	lipopolysaccharide
LC–MS	liquid chromatography–mass spectrometry
miRNAs	microRNAs
MAPK	mitogen-activated protein kinase
MDSCs	myeloid-derived suppressor cells
PLIN3	perilipin 3
PMN-MDSCs	polymorphonuclear MDSCs
*P. gingivalis*	*Porphyromonas gingivalis*
PPI	proton pump inhibitor
ROS	reactive oxygen species
Tregs	regulatory T cells
SBAs	secondary bile acids
SCFAs	short-chain fatty acids
SMOX	spermine oxidase
*S. anginosus*	*Streptococcus anginosus*
TLRs	Toll-like receptors
TMAO	trimethylamine N-oxide
TANs	tumor-associated neutrophils
T4SS	type IV secretion system

## References

[1] Bray F., Laversanne M., Sung H., Ferlay J., Siegel R.L., Soerjomataram I., Jemal A. (2024). Global Cancer Statistics 2022: GLOBOCAN Estimates of Incidence and Mortality Worldwide for 36 Cancers in 185 Countries. CA Cancer J. Clin..

[2] Yan X., Lei L., Li H., Cao M., Yang F., He S., Zhang S., Teng Y., Li Q., Xia C. (2023). Stomach Cancer Burden in China: Epidemiology and Prevention. Chin. J. Cancer Res..

[3] Akbari A., Ashtari S., Tabaiean S.P., Mehrdad-Majd H., Farsi F., Shojaee S., Agah S. (2022). Overview of Epidemiological Characteristics, Clinical Features, and Risk Factors of Gastric Cancer in Asia-pacific Region. Asia-Pac. J. Clin. Oncol..

[4] Zhou C.-B., Zhao L.-C., Qin Y., Yu J., Li W., Feng Q., Tong X., Abuduaini R., Lu S.-Y., Tang H. (2026). *Streptococcus anginosus*-Derived Methionine Promotes Gastric Cancer Progression. Gut.

[5] Mamun T.I., Younus S., Rahman M.H. (2024). Gastric Cancer—Epidemiology, Modifiable and Non-Modifiable Risk Factors, Challenges and Opportunities: An Updated Review. Cancer Treat. Res. Commun..

[6] Wizenty J., Sigal M. (2025). *Helicobacter pylori*, Microbiota and Gastric Cancer—Principles of Microorganism-Driven Carcinogenesis. Nat. Rev. Gastroenterol. Hepatol..

[7] Xia M., Lei L., Zhao L., Xu W., Zhang H., Li M., Hu J., Cheng R., Hu T. (2025). The Dynamic Oral–Gastric Microbial Axis Connects Oral and Gastric Health: Current Evidence and Disputes. npj Biofilms Microbiomes.

[8] Park S.-Y., Hwang B.-O., Lim M., Ok S.-H., Lee S.-K., Chun K.-S., Park K.-K., Hu Y., Chung W.-Y., Song N.-Y. (2021). Oral–Gut Microbiome Axis in Gastrointestinal Disease and Cancer. Cancers.

[9] Xiao X., Zhang X., Wang J., Liu Y., Yan H., Xing X., Yang J. (2024). Proton Pump Inhibitors Alter Gut Microbiota by Promoting Oral Microbiota Translocation: A Prospective Interventional Study. Gut.

[10] Takahashi N., Saito K., Schachtele C.F., Yamada T. (1997). Acid Tolerance and Acid-Neutralizing Activity of *Porphyromonas gingivalis*, *Prevotella intermedia* and *Fusobacterium nucleatum*. Oral Microbiol. Immunol..

[11] Ali Mohammed M.M., Pettersen V.K., Nerland A.H., Wiker H.G., Bakken V. (2021). Label-Free Quantitative Proteomic Analysis of the Oral Bacteria *Fusobacterium nucleatum* and *Porphyromonas gingivalis* to Identify Protein Features Relevant in Biofilm Formation. Anaerobe.

[12] Li X., Zhang S., Sheng H., Zhen Y., Wu B., Li Z., Chen D., Zhou H. (2025). Oral *Fusobacterium nucleatum* Resists the Acidic pH of the Stomach Due to Membrane Erucic Acid Synthesized via Enoyl-CoA Hydratase-Related Protein FnFabM. J. Oral Microbiol..

[13] Fu K., Cheung A.H.K., Wong C.C., Liu W., Zhou Y., Wang F., Huang P., Yuan K., Coker O.O., Pan Y. (2024). *Streptococcus anginosus* Promotes Gastric Inflammation, Atrophy, and Tumorigenesis in Mice. Cell.

[14] Gao X.-F., Zhang C.-G., Huang K., Zhao X.-L., Liu Y.-Q., Wang Z.-K., Ren R.-R., Mai G.-H., Yang K.-R., Chen Y. (2025). An Oral Microbiota-Based Deep Neural Network Model for Risk Stratification and Prognosis Prediction in Gastric Cancer. J. Oral Microbiol..

[15] You H.S., Park J.Y., Seo H., Kim B.J., Kim J.G. (2024). Increasing Correlation between Oral and Gastric Microbiota during Gastric Carcinogenesis. Korean J. Intern. Med..

[16] Kamali N., Talebi Bezmin Abadi A., Rahimi F., Forootan M. (2025). *Fusobacterium nucleatum* Confirmed in Gastric Biopsies of Patients without *Helicobacter pylori*. BMC Res. Notes.

[17] Wang B., Deng J., Donati V., Merali N., Frampton A.E., Giovannetti E., Deng D. (2024). The Roles and Interactions of *Porphyromonas gingivalis* and *Fusobacterium nucleatum* in Oral and Gastrointestinal Carcinogenesis: A Narrative Review. Pathogens.

[18] Zhang T., Li Y., Zhai E., Zhao R., Qian Y., Huang Z., Liu Y., Zhao Z., Xu X., Liu J. (2025). Intratumoral *Fusobacterium nucleatum* Recruits Tumor-Associated Neutrophils to Promote Gastric Cancer Progression and Immune Evasion. Cancer Res..

[19] Muñoz-Medel M., Pinto M.P., Goralsky L., Cáceres M., Villarroel-Espíndola F., Manque P., Pinto A., Garcia-Bloj B., de Mayo T., Godoy J.A. (2024). *Porphyromonas gingivalis*, a Bridge between Oral Health and Immune Evasion in Gastric Cancer. Front. Oncol..

[20] Liu D., Liu S., Liu J., Miao L., Zhang S., Pan Y. (2021). sRNA23392 Packaged by *Porphyromonas gingivalis* Outer Membrane Vesicles Promotes Oral Squamous Cell Carcinomas Migration and Invasion by Targeting Desmocollin-2. Mol. Oral Microbiol..

[21] Liang W., Zhou Z., Gao Q., Zhu Z., Zhu J., Lin J., Wen Y., Qian F., Wang L., Zhai Y. (2024). Tumor-Derived *Prevotella intermedia* Aggravates Gastric Cancer by Enhancing Perilipin 3 Expression. Cancer Sci..

[22] Lei L., Zhao L.-Y., Cheng R., Zhang H., Xia M., Chen X.-L., Kudriashov V., Liu K., Zhang W.-H., Jiang H. (2024). Distinct Oral-Associated Gastric Microbiota and *Helicobacter pylori* Communities for Spatial Microbial Heterogeneity in Gastric Cancer. Msystems.

[23] Marasco G., Colecchia L., Salvi D., Bruni A., Capelli C., Dajti E., Barbaro M.R., Cremon C., Stanghellini V., Barbara G. (2025). The Role of Microbiota in Upper Gastrointestinal Cancers. Cancers.

[24] Cheung M.K., Tong S.L.Y., Wong M.C.S., Chan J.Y.K., Ip M., Hui M., Lai C.K.C., Ng R.W.Y., Ho W.C.S., Yeung A.C.M. (2023). Extent of Oral–Gut Transmission of Bacterial and Fungal Microbiota in Healthy Chinese Adults. Microbiol. Spectrum.

[25] Kerstens R., Ng Y.Z., Pettersson S., Jayaraman A. (2024). Balancing the Oral-Gut-Brain Axis with Diet. Nutrients.

[26] Huang L., Jiang C., Yan M., Wan W., Li S., Xiang Z., Wu J. (2024). The Oral-Gut Microbiome Axis in Breast Cancer: From Basic Research to Therapeutic Applications. Front. Cell. Infect. Microbiol..

[27] Otálora-Otálora B.A., López-Rivera J.J., Aristizábal-Guzmán C., Isaza-Ruget M.A., Álvarez-Moreno C.A. (2023). Host Transcriptional Regulatory Genes and Microbiome Networks Crosstalk through Immune Receptors Establishing Normal and Tumor Multiomics Metafirm of the Oral-Gut-Lung Axis. Int. J. Mol. Sci..

[28] Lou F., Luo S., Kang N., Yan L., Long H., Yang L., Wang H., Liu Y., Pu J., Xie P. (2024). Oral Microbiota Dysbiosis Alters Chronic Restraint Stress-Induced Depression-like Behaviors by Modulating Host Metabolism. Pharmacol. Res..

[29] Tao K., Yuan Y., Xie Q., Dong Z. (2024). Relationship between Human Oral Microbiome Dysbiosis and Neuropsychiatric Diseases: An Updated Overview. Behav. Brain Res..

[30] Chen X.-H., Wang A., Chu A.-N., Gong Y.-H., Yuan Y. (2019). Mucosa-Associated Microbiota in Gastric Cancer Tissues Compared with Non-Cancer Tissues. Front. Microbiol..

[31] Matoba R., Iijima H., Sakamoto Y., Kawabata R., Ishiguro A., Akamaru Y., Kito Y., Yabusaki H., Matsuyama J., Takahashi M. (2022). Abstract 5957: Molecular Characteristics of Gut Microbiota in Patients with GC: The DELIVER Trial (JACCRO GC-08). Cancer Res..

[32] Zhang Y., Shen J., Shi X., Du Y., Niu Y., Jin G., Wang Z., Lyu J. (2021). Gut Microbiome Analysis as a Predictive Marker for the Gastric Cancer Patients. Appl. Microbiol. Biotechnol..

[33] Zhang R., Wu Y., Ju W., Wang S., Liu Y., Zhu H. (2024). Gut Microbiome Alterations during Gastric Cancer: Evidence Assessment of Case-Control Studies. Front. Microbiol..

[34] Zeng R., Gou H., Lau H.C.H., Yu J. (2024). Stomach Microbiota in Gastric Cancer Development and Clinical Implications. Gut.

[35] Ji J., Jung S. (2024). PredCMB: Predicting Changes in Microbial Metabolites Based on the Gene-Metabolite Network Analysis of Shotgun Metagenome Data. Bioinformatics.

[36] Ito N., Tsujimoto H., Ueno H., Xie Q., Shinomiya N. (2020). *Helicobacter pylori*-Mediated Immunity and Signaling Transduction in Gastric Cancer. J. Clin. Med..

[37] Fu W., Han X., Hao X., Zhang J., Zhang H., Ma C., Xu M., Zhang J., Ding S. (2025). Dynamic Changes of Host Immune Response during *Helicobacter pylori*-Induced Gastric Cancer Development. Clin. Exp. Immunol..

[38] Kiga K., Mimuro H., Suzuki M., Shinozaki-Ushiku A., Kobayashi T., Sanada T., Kim M., Ogawa M., Iwasaki Y.W., Kayo H. (2014). Epigenetic Silencing of miR-210 Increases the Proliferation of Gastric Epithelium during Chronic *Helicobacter pylori* Infection. Nat. Commun..

[39] Liu M., Hu Z., Wang C., Zhang Y. (2023). The TLR/MyD88 Signalling Cascade in Inflammation and Gastric Cancer: The Immune Regulatory Network of *Helicobacter pylori*. J. Mol. Med..

[40] Lai J., Angulmaduwa S., Kim M.-A., Kim A., Tissera K., Cho Y.-J., Cha J.-H. (2024). Influence of *oipA* Phase Variation on Virulence Phenotypes Related to Type IV Secretion System in *Helicobacter pylori*. Helicobacter.

[41] Roberts J.R., Tran S.C., Frick-Cheng A.E., Bryant K.N., Okoye C.D., McDonald W.H., Cover T.L., Ohi M.D. (2024). Subdomains of the *Helicobacter pylori* Cag T4SS Outer Membrane Core Complex Exhibit Structural Independence. Life Sci. Alliance.

[42] Son Y.S., Kwon Y.H., Lee M.-S., Kwon O., Jeong Y.-J., Mun S.J., Jeon S., Park J.H., Han M.-H., Bae J.-S. (2025). *Helicobacter pylori* VacA-Induced Mitochondrial Damage in the Gastric Pit Cells of the Antrum and Therapeutic Rescue. Biomaterials.

[43] Chen D., Wu L., Liu X., Wang Q., Gui S., Bao L., Wang Z., He X., Zhao Y., Zhou J. (2024). *Helicobacter pylori* CagA Mediated Mitophagy to Attenuate the NLRP3 Inflammasome Activation and Enhance the Survival of Infected Cells. Sci. Rep..

[44] Zheng H., Zhang T., Zhang J., Ning J., Fu W., Wang Y., Shi Y., Wei G., Zhang J., Chen X. (2024). AUF1-Mediated Inhibition of Autophagic Lysosomal Degradation Contributes to CagA Stability and *Helicobacter pylori*-Induced Inflammation. Gut Microbes.

[45] Liu S., Dai J., Lan X., Fan B., Dong T., Zhang Y., Han M. (2021). Intestinal Bacteria Are Potential Biomarkers and Therapeutic Targets for Gastric Cancer. Microb. Pathogen..

[46] Navashenaq J.G., Shabgah A.G., Banach M., Jamialahmadi T., Penson P.E., Johnston T.P., Sahebkar A. (2022). The Interaction of *Helicobacter pylori* with Cancer Immunomodulatory Stromal Cells: New Insight into Gastric Cancer Pathogenesis. Semin. Cancer Biol..

[47] Miao Y., Tang H., Zhai Q., Liu L., Xia L., Wu W., Xu Y., Wang J. (2022). Gut Microbiota Dysbiosis in the Development and Progression of Gastric Cancer. J. Oncol..

[48] Peng Y.-L., Wang S.-H., Zhang Y.-L., Chen M.-Y., He K., Li Q., Huang W.-H., Zhang W. (2024). Effects of Bile Acids on the Growth, Composition and Metabolism of Gut Bacteria. npj Biofilms Microbiomes.

[49] Zhang M., Zhong J., Shen Y., Song Z. (2025). Crosstalk between Bile Acids and Gut Microbiota: A Potential Target for Precancerous Lesions of Gastric Cancer. Front. Pharmacol..

[50] Szklenarik G., Kiraly P., Szegvari G., Dora D., Lohinai Z. (2024). Predicting Cancer-Related Mycobiome Aspects in Gastrointestinal Cancers: A Systematic Review. Front. Med..

[51] Gao M., Wu Y., Chen Y., Hao T., Sun X., Ji J. (2026). Disruption of Signalling Pathways Induced by HBV HBx, HCV Core, and EBV LMP1 in Gastric Cancer. Rev. Med. Virol..

[52] Angerilli V., Gasparello J., Collesei A., Ceccon C., Bergamo F., Sabbadin M., Parente P., Vanoli A., Niero M., Luchini C. (2025). Epstein-Barr Virus-Associated Gastric Cancer: A Histopathologic Study with Comprehensive Molecular Profiling. Mod. Pathol..

[53] Yang J., Liu Z., Zeng B., Hu G., Gan R. (2020). Epstein-Barr virus-associated gastric cancer: A distinct subtype. Cancer Lett..

[54] Liu J., Tian R., Sun C., Guo Y., Dong L., Li Y., Song X. (2023). Microbial Metabolites Are Involved in Tumorigenesis and Development by Regulating Immune Responses. Front. Immunol..

[55] Ahmad F., Saha P., Singh V., Wahid M., Mandal R.K., Nath Mishra B., Fagoonee S., Haque S. (2023). Diet as a Modifiable Factor in Tumorigenesis: Focus on Microbiome-Derived Bile Acid Metabolites and Short-Chain Fatty Acids. Food Chem..

[56] Cong J., Liu P., Han Z., Ying W., Li C., Yang Y., Wang S., Yang J., Cao F., Shen J. (2024). Bile Acids Modified by the Intestinal Microbiota Promote Colorectal Cancer Growth by Suppressing CD8^+^ T Cell Effector Functions. Immunity.

[57] Yin T., Zhang X., Xiong Y., Li B., Guo D., Sha Z., Lin X., Wu H. (2024). Exploring Gut Microbial Metabolites as Key Players in Inhibition of Cancer Progression: Mechanisms and Therapeutic Implications. Microbiol. Res..

[58] McNamara K.M., Sierra J.C., Latour Y.L., Hawkins C.V., Asim M., Williams K.J., Barry D.P., Allaman M.M., Zagol-Ikapitte I., Luis P.B. (2025). Spermine Oxidase Promotes *Helicobacter pylori*-Mediated Gastric Carcinogenesis through Acrolein Production. Oncogene.

[59] Jia D., Kuang Z., Wang L. (2024). The Role of Microbial Indole Metabolites in Tumor. Gut Microbes.

[60] Zhou Y., Zhang Y., Jin S., Lv J., Li M., Feng N. (2024). The Gut Microbiota Derived Metabolite Trimethylamine N-Oxide: Its Important Role in Cancer and Other Diseases. Biomed. Pharmacother..

[61] Bockerstett K.A., DiPaolo R.J. (2017). Regulation of Gastric Carcinogenesis by Inflammatory Cytokines. Cell. Mol. Gastroenterol. Hepatol..

[62] Zhao W., Liu M., Zhang M., Wang Y., Zhang Y., Wang S., Zhang N. (2021). Effects of Inflammation on the Immune Microenvironment in Gastric Cancer. Front. Oncol..

[63] Nwabo Kamdje A.H., Tagne Simo R., Fogang Dongmo H.P., Bidias A.R., Masumbe Netongo P. (2023). Role of Signaling Pathways in the Interaction between Microbial, Inflammation and Cancer. Holist. Integr. Oncol..

[64] Dooyema S.D.R., Noto J.M., Wroblewski L.E., Piazuelo M.B., Krishna U., Suarez G., Romero-Gallo J., Delgado A.G., Peek R.M. (2022). *Helicobacter pylori* Actively Suppresses Innate Immune Nucleic Acid Receptors. Gut Microbes.

[65] Zhu X., Zhang X., Shen J., Zheng S., Li H., Han B., Zhang C., Chen M., Sun Q., Wu J. (2024). Gut Microbiota-Dependent Modulation of Pre-Metastatic Niches by Jianpi Yangzheng Decoction in the Prevention of Lung Metastasis of Gastric Cancer. Phytomed. Int. J. Phytother. Phytopharm..

[66] Bintintan V., Burz C., Pintea I., Muntean A., Deleanu D., Lupan I., Samasca G. (2024). The Importance of Extracellular Vesicle Screening in Gastric Cancer: A 2024 Update. Cancers.

[67] Chang C.-P., Hu M.-H., Hsiao Y.-P., Wang Y.-C. (2020). ST2 Signaling in the Tumor Microenvironment. Adv. Exp. Med. Biol..

[68] Nie S., Wang A., Chen X., Gong Y., Yuan Y. (2023). Microbial-Related Metabolites May Be Involved in Eight Major Biological Processes and Represent Potential Diagnostic Markers in Gastric Cancer. Cancers.

[69] Gou H., Zeng R., Lau H.C.H., Yu J. (2024). Gut Microbial Metabolites: Shaping Future Diagnosis and Treatment against Gastrointestinal Cancer. Pharmacol. Res..

[70] Yang Y., Huang Y., Lin W., Liu J., Chen X., Chen C., Yu X., Teng L. (2022). Host miRNAs-Microbiota Interactions in Gastric Cancer. J. Transl. Med..

[71] Chen Z., Jin D., Hu J., Guan D., Bai Q., Gou Y. (2025). Microbiota and Gastric Cancer: From Molecular Mechanisms to Therapeutic Strategies. Front. Cell. Infect. Microbiol..

[72] Ishikawa H., Aoki R., Mutoh M., Ishiguro S., Tanaka T., Miyoshi N., Miyamoto S., Hamoya T., Yoshida N., Wakabayashi K. (2025). Contribution of Colibactin-Producing *Escherichia Coli* to Colonic Carcinogenesis. Egastroenterology.

[73] Kim M.-J., Kim H.-N., Jacobs J.P., Yang H.-J. (2024). Combined DNA Methylation and Gastric Microbiome Marker Predicts *Helicobacter pylori*-Negative Gastric Cancer. Gut Liver.

[74] Wang L., Xin Y., Zhou J., Tian Z., Liu C., Yu X., Meng X., Jiang W., Zhao S., Dong Q. (2020). Gastric Mucosa-Associated Microbial Signatures of Early Gastric Cancer. Front. Microbiol..

[75] Zaramella A., Arcidiacono D., Duci M., Benna C., Pucciarelli S., Fantin A., Rosato A., De Re V., Cannizzaro R., Fassan M. (2024). Predictive Value of a Gastric Microbiota Dysbiosis Test for Stratifying Cancer Risk in Atrophic Gastritis Patients. Nutrients.

[76] Radhika T., Gopalakrishnan S., Muthukumar R.S., Arulpari M., Kumar B.S., Hari R., Jeyaraman M., Jeddy N. (2023). Role of Serum and Salivary microRNAs as Diagnostic Biomarkers in Gastric Cancer. J. Orofac. Sci..

[77] Rahimi A.M., Nabavizadeh F., Ashabi G., Halimi S., Rahimpour M., Vahedian J., Panahi M. (2021). Probiotic *Lactobacillus rhamnosus* Supplementation Improved Capecitabine Protective Effect against Gastric Cancer Growth in Male BALB/c Mice. Nutr. Cancer.

[78] Linsalata M., Cavallini A., Messa C., Orlando A., Refolo M.G., Russo F. (2010). *Lactobacillus rhamnosus* GG Influences Polyamine Metabolism in HGC-27 Gastric Cancer Cell Line: A Strategy toward Nutritional Approach to Chemoprevention of Gastric Cance. Curr. Pharm. Des..

[79] Raoul P., Maccauro V., Cintoni M., Scarpellini E., Ianiro G., Gasbarrini A., Mele M.C., Rinninella E. (2024). Microbiota-Gastric Cancer Interactions and the Potential Influence of Nutritional Therapies. Int. J. Mol. Sci..

[80] Qian L., Gao R., Huang J., Qin H. (2019). Supplementation of Triple Viable Probiotics Combined with Dietary Intervention Is Associated with Gut Microbial Improvement in Humans on a High-Fat Diet. Exp. Ther. Med..

[81] Jia K., Chen Y., Dai D., Xie Y., Peng H., Cao Y., Zou H., Qiu C., Tan Y., Zhang X. (2026). Impact of *Helicobacter pylori* Infection on Gut and Intratumoral Microbiome and Its Association with Immunotherapy Response in Gastrointestinal Cancer. BMC Med..

[82] Zhu Z., Huang J., Zhang Y., Hou W., Chen F., Mo Y.Y., Zhang Z. (2024). Landscape of tumoral ecosystem for enhanced anti-PD-1 immunotherapy by gut *Akkermansia muciniphila*. Cell Rep..

